# Intranasal dexmedetomidine and intranasal ketamine association allows shorter induction time for pediatric sedation compared to intranasal dexmedetomidine and oral midazolam

**DOI:** 10.1186/s13052-021-01196-0

**Published:** 2022-01-10

**Authors:** Francesca Cossovel, Andrea Trombetta, Augusto Ramondo, Guglielmo Riccio, Luca Ronfani, Alessia Saccari, Giorgio Cozzi, Egidio Barbi

**Affiliations:** 1 Department of Medical, Surgical and Health Sciences, University of Trieste, Via dell’Istria 65/1, 34137 Trieste, Italy; 2 Institute for Maternal and Child Health IRCCS “Burlo Garofolo”, Trieste, Italy

**Keywords:** Pediatric procedural sedation, Intranasal dexmedetomidine, Intranasal ketamine, Oral midazolam

## Abstract

**Background:**

Non-painful diagnostic procedures require an inactive state for a prolonged time, so that sedation is often needed in younger children to perform the procedures. Our standard of care in this setting consists of the association between oral midazolam (0.5 mg/kg) and intranasal dexmedetomidine (4 mcg/kg). One of the limits of this approach is that the onset of action is quite delayed (up to 55 min) and poorly predictable. We chose to compare this association with intranasal-ketamine and intranasal-dexmedetomidine.

**Methods:**

This is a “pre-post” study. The study population included the first forty children receiving sedation with the “new” combination intranasal ketamine (3 mg/kg) and intranasal dexmedetomidine (4 mcg/kg) compared to a historical cohort including the last forty children receiving sedation with our standard of care combination of intranasal dexmedetomidine (4mcg/kg) and oral midazolam (0,5 mg/kg).

**Results:**

The association intranasal dexmedetomidine and intranasal ketamine allowed for a significantly shorter sedation induction time than the combination intranasal dexmedetomidine and oral midazolam (13,5 min versus 35 min). Both group’s cumulative data showed a correlation between age and sedation effectiveness, with younger children presenting a higher success rate and shorter induction time (p 0,001). Conclusions: This study suggests that the ketamine and dexmedetomidine intranasal association may have a shorter onset of action when compared to intranasal dexmedetomidine and oral midazolam.

## Introduction

Non-painful diagnostic procedures require an inactive state for a prolonged time and sedation is often needed for infants and young children [1].

Among the different possible strategies in non-painful procedures, two recent studies investigated the efficacy of the combination of intranasal dexmedetomidine (2 mcg/kg) and intranasal ketamine (1 mg/kg) for pediatric procedural sedation. These series showed that over 90% of patients completed the procedure without a sedative rescue treatment [2, 3]. Our standard of care in this setting consists of the association between oral midazolam (0.5 mg/kg) and intranasal dexmedetomidine (4 mcg/kg). This approach has a rather long induction time as a limit, with an average of 35 min and peaks up to 55 min before adequate sedation is reached [4].

We recently adopted a protocol with the combination of intranasal ketamine and intranasal dexmedetomidine, in the attempt to reduce the induction time and to improve the number of procedures achieved without need of rescue treatment.

## Methods

This comparative observational study was carried out at the tertiary-level, university teaching, Institute for Maternal and Child Health IRCCS Burlo Garofolo of Trieste, Italy, between January and June 2020. The Institutional Review Board approved the study, IRB 04/20.

The medical records of the consecutively enrolled last 42 children sedated at our Institution with the combination of intranasal dexmedetomidine and oral midazolam (DexMid group) and the first 43 children sedated with the combination of intranasal ketamine and intranasal dexmedetomidine (KetoDex group) were retrospectively revised. We included patients needing procedural sedation from 0 to 18 years of age. Exclusion criteria were: sedation for painful procedures, sedation with prior adverse reactions to midazolam, ketamine or dexmedetomidine, sedation with general risk factors contraindicating these drugs. These were hypotension and history of paradoxical reactions for midazolam; age below 3 months for ketamine, and hypotension, hypertension, bradycardia and use of beta-blockers or digoxin for dexmedetomidine. Further exclusion criteria were blood/mucous obstructing the nasal passage, non-compliance with fasting for elective procedures (2 h for clear liquids, 4 h for breast milk, 8 h for solids), airway malformations or history of significant airway obstruction (i.e. heavy snoring, obstructive sleep apnoea, macroglossia, micrognathia).

A sedation procedure was defined as successful when it could be performed without need of rescue drugs or physical restraint.

Two children in the DexMid group and 3 patients in the KetoDex group were excluded from the analysis due to missing data.

Children among the KetoDex group were sedated with 3 mg/kg of intranasal ketamine (50 mg/ml) and 4mcg/kg of diluted galenic intranasal dexmedetomidine (50mcg/ml).

Children among the DexMid group were sedated with 4mcg/kg of diluted galenic intranasal dexmedetomidine (50mcg/ml^-1^) and 0.5 mg/kg of oral midazolam (5 mg/ml).

The choice of using diluted galenic dexmedetomidine (50mcg/ml) in our Institute is arbitrarily based on the intended goal to avoid overdosage risks in younger children based on our experience with newborns [5].

All children enrolled in the study were sedated for non-painful procedures.

Children received a topical application of Eutectic Mixture of Local Anesthetics (EMLA)-cream to facilitate intravenous cannulation if needed for rescue sedatives or by the procedure (eg, MRI with contrast medium). Intranasal sedatives were administered using a mucosal atomization device. Patient monitoring during sedation includes the constant recording of oxygen saturation and heart rate. Post-procedural monitoring was performed in the paediatric ward and continued until the patient was awake with a minimum Aldrete score of 9 points and could assume clear liquids [6]. Sedation adverse effects were classified by using the Tracking and Reporting Outcomes Of Procedural Sedation (TROOPS) [7].

Data collection included demographic variables, American Society of Anesthesiology (ASA) score, drug dosages, the type of procedure and its duration, the time required to achieve a Ramsay sedation score of at least 4, the time to recover from sedation, the sedation-related adverse events, the need for rescue treatment or medical support.

The study’s primary outcome was to evaluate the induction time of the two combinations of sedatives.

Secondary outcomes included the procedures’ success rate, the time to recover from sedation and the number and type of adverse events.

Continuous variables are reported as medians and interquartile ranges (IQR), or as means and standard deviations (SD). Categorical data are reported as numbers and percentages. We evaluated the correlations between the two groups using the Chi-square test, Mann-Whitney test, and Fisher exact test for parametric correlations and Rho di Spearman for non-parametric correlations. A *p*-value < 0.05 was considered for statistical significance. Data were analyzed with Stata/IC 14.2 (StataCorp LP, College Station, TX, USA).

## Results

The study population included 80 children, 40 of whom in the KetoDex group and 40 in the DexMid group (Table 1). The combination of intranasal ketamine and intranasal dexmedetomidine allowed for a significantly shorter sedation induction time than the combination intranasal dexmedetomidine and oral midazolam (Table 1).

**Table 1:** Characteristics of the study population, study outcomes and type of procedure

	KetoDex group	DexMid group	
	***n*** = 40	***n*** = 40	p
**Characteristics of the study population**			
Gender male, n (%)	24 (60.0%)	18 (45.0%)	0.18
Weight (Kg), median (IQR)	12.5 (11.0–17.0)	14.9 (11.5–18.0)	0.29
Age (months), median (IQR)	29.5 (20.0–49.5)	45.5 (31.5–56.5)	0.05
0–1 years (%)	2 (5%)	7 (17.5%)	
1–2 years (%)	12 (30%)	1 (2.5%)	
2–3 years (%)	12 (30%)	4 (10%)	
3–4 years (%)	4 (10%)	12 (30%)	
4–5 years (%)	5 (12.5%)	7 (17.5%)	
5–6 years (%)	1 (2.5%)	4 (10%)	
6–7 years (%)	1 (2.5%)	3 (7.5%)	
7–8 years (%)	1 (2.5%)	0	
9–10 years (%)	1 (2.5%)	0	
10–11 years (%)	1 (2.5%)	0	
11–12 years (%)	0	1 (2.5%)	
14–15 years (%)	0	1 (2.5%)	
Comorbidities	5 (2 tonsillar hyprtrophy grade III, 1 NF1, 1 macrocephaly, 1 prematurity with bronchial dysplasia)		
**Study outcomes**			
Procedures’ success rate (%)	31 (78.5%)	33 (82.5%)	OR 0,73 (0.24–2.2)
Induction time (minutes), median (IQR)	13.5 (10.0–20.0)	35.0 (21.3–44.5)	< 0.001
Recovery time (minutes), median (IQR)	72.5 (60.3–103.0)	71.5 (53.0–90.8)	0.32
Need for rescue sedative, n (%)	9 (22.5%)	7 (17.5%)	0.58
Adverse events, n (%)	2 (5.0%)	4 (10.0%)	0.68
Type of Adverse events (%)^a^	Vomiting (50%), self resolving desaturation (50%)	Self resolving desaturations (100%)	
**Type of procedure**			
Magnetic Resonance Imaging	28 (70%)	40 (100%)	NA
Renal Scintigraphy	4 (10%)	0 (0%)	NA
Computer Tomography	3 (7,5%)	0 (0%)	NA
Electroencephalography	1 (2,5%)	0 (0%)	NA
Echocardiography	2 (5%)	0 (0%)	NA
Auditory Brainstem Response	2 (5%)	0 (0%)	NA

*NF1* neurofibromatosis type 1, *NA* not applicable

^a^According to the Tracking and Reporting Outcomes Of Procedural Sedation

**Fig. 1:**
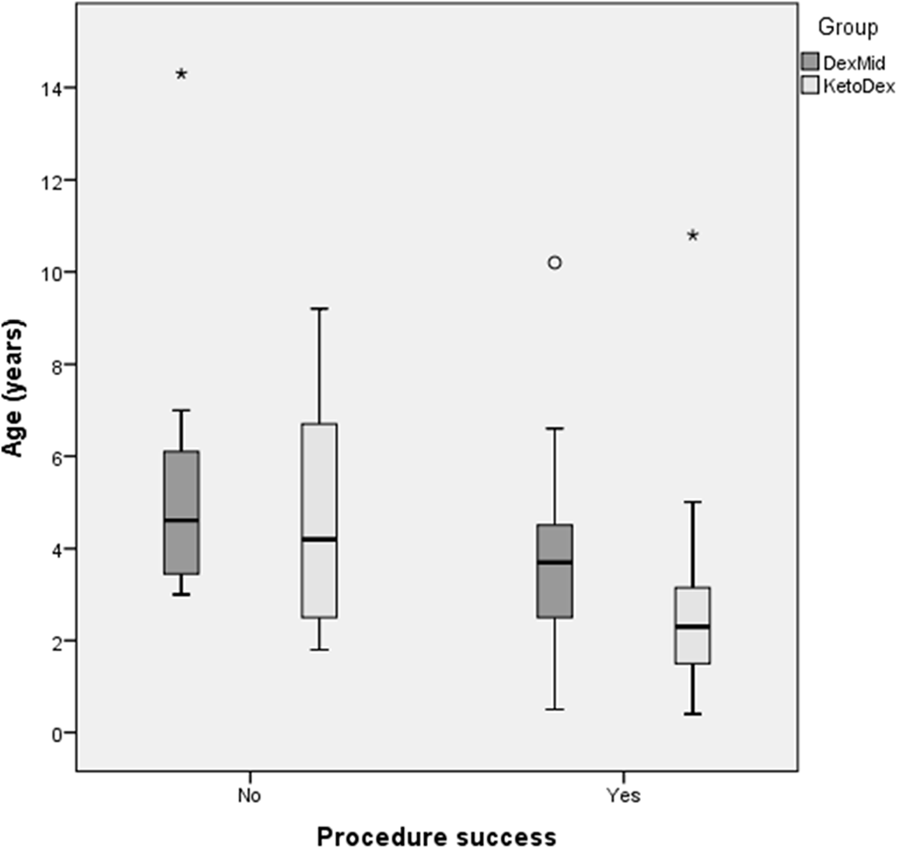
Correlation between ages and sedation effectiveness in KetoDex and DexMid groups: younger children below one year of age present a higher success rate and shorter induction time (p 0,001)

Both group’s cumulative data revealed a correlation between the sedation effectiveness and the age of participants, being the higher success rate related to a lower mean age (*p* = 0.005) and shorter induction time (p 0,001). The procedure success was not significantly different among the two groups (Fig. 1). According to the TROOPS system, adverse events were few and mild, consisting in 1 episode of vomit in the KetoDex group and 5 self-resolving mild desaturations, 4 in the DexMid group and 1 in the KetoDex group. None of these episodes required an unplanned intervention. This was true also for the KetoDex group, which resulted in a higher percentage of children with comorbidity and higher ASA scores.

## Discussion

In this study the combination of intranasal ketamine and intranasal dexmedetomidine allowed for a significantly shorter sedation induction time than the combination intranasal dexmedetomidine and oral midazolam. Furthermore, the ketamine-dexmedetomidine association showed a similar procedure success and safety drug profile, even in patients with higher ASA score. Although these results could be in part expected from the faster route of administration (oral midazolam vs intranasal ketamine) [8] and from the different action of drugs (anxiolytic- midazolam vs sedative- ketamine) [9], the last issue may suggest that this combination is safer than the one with oral midazolam, but far more data are needed in these perspectives.

Remarkably, when compared with other studies in the literature, our data on the success rate of the combination of intranasal ketamine and intranasal dexmedetomidine showed a lower percentage of successful procedures, despite the higher dosages used in order to identify a possibly more effective dose, 3 mg/kg and 4 mcg/kg versus 1 mg/kg and 2 mcg/kg respectively [2, 3]. These different results may be due to the use of diluted galenic dexmedetomidine (50mcg/ml) in our Institute, versus the 100 mcg/ml strength of the standard iv drug, suggesting a lower effectiveness of a more diluted formulation. Further studies may address this issue.

The study’s main limits are its retrospective nature and the limited number of patients enrolled.

On the other hand, this is the first study investigating the difference in sedation onset between these two approaches and the relationship between age and sedation efficacy in the KetoDex versus DexMid association setting.

## Conclusion

This study suggests that the ketamine and dexmedetomine intranasal association has a shorter onset of action when compared to intranasal dexmedetomidine and oral midazolam. This data could be relevant form a pragmatic perspective for better planning of procedures. Prospective studies are needed to better define these issues.

## Data Availability

All data generated or analysed during this study are included in this published article.

## Abbreviations

ASA: American Society of Anesthesiology
DexMid: Intranasal dexmedetomidine and oral midazolam
EMLA: Eutectic Mixture of Local Anesthetics
IQR: Interquartile range
KetoDex group: Intranasal ketamine and intranasal dexmedetomidine
SD: standard deviation
